# Good brace compliance, reduced curve progression, and surgical rates in patients with idiopathic scoliosis

**DOI:** 10.1186/1748-7161-8-S1-O43

**Published:** 2013-06-03

**Authors:** JI Brox, JE Lange, RB Gunderson, H Steen

**Affiliations:** 1 Oslo University Hospital, Oslo, Norway

## Aim

To examine the association between brace compliance and outcome.

## Methods

495 (457 female) patients with late onset juvenile and adolescent idiopathic scoliosis were examined prospectively, before bracing, and at least 2 years after brace weaning. One spine surgeon examined all patients. 381 (353 females) answered a standardised questionnaire, and 355 had radiological examination after median 24 years. Compliance was defined as brace wear > 20 hours daily until weaning. Main outcomes were curve progression and surgery.

## Results

At weaning, 76/389 compliers and 59/106 non-compliers had curve progression ï‚³ 6° (OR: 5.2; 95 % CI: 3.3 to 8.2). At long-term, the numbers were 68/284 and 46/71 (OR: 5.8; 95% CI: 3.3 to 10.2), and 10/284 versus 17/71 had been operated (OR: 8.6; 95 % CI: 3.7 to 19.9).

## Conclusion

We conclude that the risk for curve progression, and surgery, are reduced in patients with good brace compliance.

## References

[B1] Rahman T Bowen JR Takemitsu M Scott C The association between brace compliance and outcome for patients with idiopathic scoliosis J Pediatr Orthop 2005 25 4 420 422 10.1097/01.bpo.0000161097.61586.bb 15958887

